# Physical function estimates change in pain following IIPT among children with chronic pain

**DOI:** 10.1111/papr.70009

**Published:** 2025-01-20

**Authors:** Mayank Seth, Katherine Bentley, Kathryn Hottinger, Kate Vieni, Anke Reineke, Pritha Dalal

**Affiliations:** ^1^ Research Department Children's Specialized Hospital Long Term Care Center Mountainside New Jersey USA; ^2^ Department of Physical Medicine and Rehabilitation Rutgers New Jersey Medical School Newark New Jersey USA; ^3^ Physiatry Children's Specialized Hospital New Brunswick New Jersey USA; ^4^ Psychology Children's Specialized Hospital New Brunswick New Jersey USA; ^5^ Physical Therapy Children's Specialized Hospital New Brunswick New Jersey USA; ^6^ Inpatient Chronic Pain Management Program Rady Children's Hospital San Diego California USA

**Keywords:** function, pain reduction, pediatric, self‐report

## Abstract

**Introduction:**

Chronic pain can negatively impact a child's quality of life. Pediatric Intensive Interdisciplinary Pain Treatment (IIPT) programs aim to improve overall functioning despite pain through various rehabilitative strategies. It is, however, unclear whether improved function corresponds to self‐reported decrease in pain levels. Hence, the purpose of this study is to examine the relationship between changes in physical function and perceived pain among children with chronic pain who have undergone inpatient IIPT.

**Materials and Methods:**

A secondary analysis of pre‐existing databases of IIPT from two different inpatient acute rehabilitation programs was carried out. Children and adolescents (*N* = 309; age = 16.2 ± 2.6; 79% females) with chronic pain who attended on average 4‐week inpatient IIPT from Nov 2011 to Jan 2023 were included. Participants completed pain intensity (Numerical Pain Rating Scale) and self‐reported function measures (Lower Extremity Functional Scale [LEFS], Upper Extremity Functional Index [UEFI], Canadian Occupational Performance Measure [COPM]‐Performance, and COPM‐Satisfaction) at admission and discharge.

**Results:**

Change in self‐reported physical function was significantly associated with change in pain from admission to discharge. After covariate adjustment, self‐reported physical function (per the LEFS, UEFI, COPM‐Performance, and COPM‐Satisfaction) explained 19.8%, 7.8%, 12.0%, and 8.6% of the variance in change in pain, respectively. These measures of self‐reported physical function further distinguished between minimal (<30%) and moderate (≥30%) pain reduction.

**Conclusions:**

Self‐reported functional gains during IIPT are associated with greater change in perceived pain. Moreover, measures of self‐reported physical function can help identify children at risk of minimal pain reduction post‐IIPT.

## INTRODUCTION

Chronic pain is characterized as pain that persists beyond the expected healing period, that is, 3 months or more.^1^, ^2^ This persistent pain may contribute to the development of long‐term movement‐related fear and fear avoidance behavior.^3^ Over time, fear‐related activity avoidance may result in a decline in overall functioning, which may further lead to adverse psychological and social outcomes.^3^ For example, children experiencing chronic pain are more likely to exhibit mental health comorbidities, such as, anxiety, depression, or diminished emotional well‐being.^4^, ^5^ Additionally, having chronic pain often leads to reduced school attendance and withdrawal from recreational and extracurricular activities.^6^ Traditional pharmacologic and acute pain treatments have shown limited success in managing pain symptoms and improving outcomes in this population.^7^, ^8^ In contrast, biopsychosocial approaches like Intensive Interdisciplinary Pain Treatment (IIPT), that incorporate rehabilitative strategies aimed at enhancing overall functioning despite pain, are considered more suitable given their broad impact.^9^ However, the extent to which enhanced function correlates with improved patient outcomes, particularly perceived pain, remains unclear. This relationship is important to understand since direct and repetitive assessments of pain are typically avoided during IIPT to prevent exacerbating negative attention toward pain.^10^

IIPT applies principles of the biopsychosocial model of care in improving overall patient functioning.^9^ Patient treatment encompasses a comprehensive focus on several domains, such as physical (including, muscle strengthening and manual exercises), emotional (including the development of effective coping strategies), or social (encouraging re‐engagement in previously avoided activities).^11^, ^12^ While pain reduction is not the primary focus of patient rehabilitation, an improvement in overall functioning may coincide with reduced perceived pain.^13^ This change in perceived pain may be largely attributed to the IIPT's emphasis on graded exposure to exercises, muscle strengthening, and the enhancement of psychosocial outcomes. Existing literature supports the positive impact of aerobic and isometric exercise training on reducing pain intensity, both in children^14^ and adults with chronic pain, with exercise‐induced hypoalgesia proposed as a potential underlying mechanism.^15^ Furthermore, ample evidence underscores the significant role of psychosocial factors, such as distress, pain catastrophizing, and active pain‐coping, in either exacerbating or ameliorating pain experiences.^16^, ^17^ Thus, the improvement of overall patient functioning following IIPT is likely associated with reduced perceived pain. Current literature, however, provides limited clinical guidance on estimating changes in pain based on an individual's functional status.

Estimating change in pain levels following IIPT is crucial for optimizing patient care and treatment outcomes. For example, if minimal pain reduction is expected post‐treatment (i.e., <30% change from admission),^18^ the clinical team can proactively explore underlying factors—whether physical or psychosocial—to optimize intervention effectiveness. Additionally, this approach helps establish realistic goals for patients, fostering a more personalized approach to care and enhancing patient engagement and satisfaction. Lastly, patient stratification based on anticipated pain levels optimizes intervention delivery, allowing judicious resource allocation and tailoring of therapeutic modalities to specific patient needs. Hence, the purpose of this study is to examine the relationship between changes in physical function and perceived pain among children with chronic pain who have undergone inpatient IIPT. We hypothesized that change in self‐reported physical function would be associated with change in perceived pain from admission to discharge. Furthermore, we hypothesize that measures of self‐reported physical function would significantly distinguish between change in pain levels.

## METHODS

The data for this secondary analysis was retrieved from previous datasets of inpatient IIPT programs at Children's Specialized Hospital (CSH) in New Brunswick, NJ and Rady Children's Hospital (RCH) in San Diego, CA. Children who received clinical care and participated in the IIPT between November 2011 to January 2023 at CSH and between May 2019 to September 2023 at RCH were eligible for inclusion if they were between the ages of 9 and 22 years, had a chronic pain diagnosis (i.e., pain lasting ≥3 months), and had previously received ineffective outpatient treatment for chronic pain. In addition to having a cognitive, neurological, or musculoskeletal problem that prevented them from participating in the program, individuals were ineligible if they had suicidal thoughts or attempted suicide within the previous 6 months. This secondary analysis was approved as exempt by local ethics committees at both hospitals.

### IIPT protocol

Participants took part in a 4‐week (average duration of stay) IIPT program at CSH or RCH that was designed to help participants change their physical sensations, catastrophic thinking, and unhealthy habits, while also enhancing their strength, endurance, and function. The typical routine at both hospitals included individual and group sessions of meditation, cognitive behavioral therapy, aqua therapy, physical therapy, and occupational therapy. Most physical and occupational therapy sessions were given individually to participants (5×/week), with a focus on endurance training, generalized strengthening, exercise education, goal‐oriented task training to improve speed and quality of movement, and isolated and functional desensitization of affected extremities of participants with allodynia. To aid patients in improving their communication, problem‐solving, and coping abilities, psychologists meet with them two to three times per week individually and co‐treated with the other therapists. Group physical and occupational therapy sessions were done once every week, involving exercise and school‐related tasks. Daily aquatic therapy group sessions emphasized increasing lower extremity weight bearing while enhancing functional strength and overall balance. Additionally, regular group meditation sessions focused on increased mindfulness and relaxation.

### Procedures

#### Participant demographics

At both hospitals, a clinical team member collected data on the patient's demographics (such as age, gender, and race), pain diagnosis, comorbidities (such as injury history and psychological diagnostic history), pain medication use, and assistive technology use.

#### Pain intensity

The Numerical Pain Rating Scale (NPRS) is a validated measure of pain intensity among children with chronic pain.^19^ The NPRS is an 11‐point scale (0–10) where “0” denotes no pain and “10” denotes worst imaginable pain. Each patient rated their pain intensity at the time of admittance to the IIPT and at discharge.

#### Function

The following patient‐reported functional outcomes were completed by participants at admittance and discharge from the IIPT program.

The Lower‐Extremity Functional scale (LEFS) is a valid and reliable measure to evaluate an individual's lower extremity function in different activities.^20^ The scale comprises 20 items, each rated on a 5‐point scale (0–4). A score of “0” represents “extreme difficulty or inability to perform the activity,” while a score of “4” indicates “no difficulty.” The total score ranges from 0 to 80, with higher scores reflecting better lower extremity function.^21^

The Upper Extremity Functional Index (UEFI) is a valid and reliable measure to evaluate an individual's upper extremity function on across a range of activities.^22^ The UEFI comprises 20 items, each rated on a 5‐point scale (0–4). A score of “0” represents “extreme difficulty or inability to perform the activity,” while a score of “4” indicates “no difficulty.”^22^ The scores of all items are summed to achieve a maximum possible score of 80. Higher UEFI scores indicate better upper extremity functional status.^22^

The Canadian Occupational Performance Measure (COPM) is a valid measure that focuses on the client's perspective to assess changes in their perceived occupational performance.^21^, ^23^ To administer the COPM, a trained Occupational Therapist conducts a semi‐structured interview with the patient, discussing activities that hold significance to them.^23^ After identifying five of the most important activities, patients rate their ability to perform each activity on a scale of 1 to 10. A score of “1” indicates “not at all able,” while “10” indicates “able to perform the activity extremely well.”^23^ The average score of the five activities provides the patient's COPM Performance rating.^23^ Furthermore, patients provide a satisfaction rating for each of the five important activities on a scale of 1 to 10. “1” represents “not at all satisfied,” while “10” indicates “extremely satisfied.”^23^ The average satisfaction score of the five activities yields the COPM Satisfaction rating.^23^ An increase in both performance and satisfaction ratings over time signifies an improvement in the patient's perception of their occupational performance.^21^, ^23^

### Statistical analysis

All statistical analysis were conducted using SPSS 28 (IBM Corp., Armonk, New York, USA). Participant characteristics, pain intensity, and functional outcomes were examined with descriptive statistics. Correlations between age, gender, history of injury, previous psychological diagnosis, change in pain intensity, and changes in functional outcomes at discharge (including change in LEFS scores, UEFI scores, COPM Performance scores, and COPM Satisfaction scores) were examined using Pearson's correlation coefficient. Separate hierarchical multiple regression was conducted for each functional outcome change score (independent variable), with change in pain intensity as the dependent measure. Age and sex (0 = male; 1 = female) were entered in Block I. Data source (1 = CSH; 2 = RCH) was entered in Block II. History of injury (0 = No; 1 = Yes) and previous psychological diagnosis (0 = No; 1 = Yes) were entered in Block III. Functional outcome measures were entered in Block IV. For all planned analysis significance was accepted at *α* ≤ 0.050.

#### Optimal thresholds

Based on prior literature, participant change in pain intensity was dichotomized as minimal change (<30%) or moderate to significant change (≥30%).^18^ Receiver operating characteristic (ROC) curves were utilized to determine each functional measures' ability to distinguish between change in pain status. The dependent measure for this analysis was the change in pain classification, with minimal change assigned a value of 0 and moderate to significant change assigned a value of 1. For each ROC model, participants' response on a functional measure at discharge was considered the independent measure. Optimal thresholds for significant models were determined using Youden's index.^24^

## RESULTS

### Participants

Overall, 446 (CSH, *n* = 371; RCH, *n* = 75) children and adolescents with chronic pain received treatment as part of the IIPT program. Of these, 119 were missing data on their pain rating at either admission or discharge, and 12 were missing data on all four of the selected functional outcomes. Hence, 315 participants were considered for the final analysis. Further residual analysis resulted in the exclusion of 6 cases, given their residuals were outliers on 3 of the 4 functional outcome measures. The demographic characteristics of the 309 participants included in the final analysis are presented in Table 1. The majority of participants were females (i.e., 79.0%) and white (i.e., 80.1%), and Amplified Musculoskeletal Pain Syndrome (AMPS) was the most reported pain diagnosis (i.e., 39.8%). While most participants did not report a history of injury (i.e., 62.7%), the majority reported having a psychological diagnosis, such as anxiety or depression (i.e., 66.6%).

**TABLE 1:** Participant demographics (*n* = 309).

Measures	
Age (years), mean (±SD)	16.2 (2.6)
Gender, frequency (%)	
Male	65 (21.0)
Female	244 (79.0)
Race, frequency (%)	
White	238 (80.1)
Black or African American	10 (3.4)
Asian	9 (3.0)
American Indian or Alaskan Native	1 (0.3)
Other or multiple races	39 (13.1)
Total valid cases	297
Pain diagnosis, frequency (%)	
CRPS	67 (21.7)
Fibromyalgia	3 (1.0)
AMPS	123 (39.8)
POTS	1 (0.3)
Chronic abdominal pain	4 (1.3)
Chronic headache	6 (1.9)
FND	8 (2.6)
Chronic pain syndrome	11 (3.6)
Other	6 (1.9)
Two pain diagnosis	56 (18.1)
Three or more pain diagnosis	24 (7.8)
History of injury, frequency (%)	
No	193 (62.7)
Yes	115 (37.3)
Total valid cases	308
Psychological diagnosis^a^, frequency (%)	
No	103 (33.4)
Yes	205 (66.6)
Total valid cases	308
Pain medication, frequency (%)	
No	159 (51.5)
Yes	150 (48.5)
Assistive technology, frequency (%)	
No	204 (66.9)
Yes	101 (33.1)
Total valid cases	309

*Note*: Total valid cases represent cases for which data was available on the measure.

Abbreviations: AMPS, Amplified Musculoskeletal Pain Syndrome; CRPS, Complex Regional Pain Syndrome; POTS, Postural Orthostatic Tachycardia Syndrome.

^a^ Diagnosis of anxiety, depression, conversion disorder, attention‐deficit hyperactivity disorder, obsessive compulsive disorder, bipolar disorder.

Mean perceived pain intensity and score on each physical function outcome at admission and discharge, along with change scores is presented in Table 2. Overall, participants demonstrated improvements across all measures. Correlations between change in pain and changes on each functional outcome are presented in Table 3. Change in pain was significantly correlated with change in LEFS (*r* = 0.47), change in UEFI (*r* = 0.32), change in COPM‐Performance (*r* = 0.36), and change in COPM‐Satisfaction (*r* = 0.32).

**TABLE 2:** Participant pain and functional outcomes at admission and discharge (*n* = 309).

Measure	Admission	Discharge	Change
NPRS (0–10)	6.8 (2.0)	4.0 (2.4)	2.8 (2.7)
LEFS (0–80)	35.9 (15.9)	64.6 (13.9)	28.7 (17.0)
Total valid cases	285	285	285
UEFI (0–80)	49.0 (15.7)	69.2 (10.1)	20.0 (14.0)
Total valid cases	218	218	214
COPM performance (1–10)	3.6 (1.3)	7.5 (1.7)	3.9 (1.8)
Total valid cases	256	252	252
COPM satisfaction (1–10)	2.6 (1.4)	7.6 (2.1)	5.0 (2.4)
Total valid cases	255	250	250

*Note*: All data are presented as mean (±SD). Total valid cases represent cases for which data was available on the measure.

Abbreviations: COPM, Canadian Occupational Performance Measure; LEFS, Lower‐Extremity Functional Scale; NPRS, Numerical Pain Rating Scale; UEFI, Upper Extremity Functional Index.

**TABLE 3:** Correlations between change in pain intensity and change in functional outcomes.

	NPRS change	LEFS change	UEFI change	COPM performance change	COPM satisfaction change
NPRS change	1				
LEFS change	0.470*	1			
UEFI change	0.318*	0.590*	1		
COPM performance change	0.361*	0.484*	0.433*	1	
COPM satisfaction change	0.320*	0.376*	0.367*	0.786*	1

*Note*: Data presented as Pearson's *r*.

Abbreviations: COPM, Canadian Occupational Performance Measure; LEFS, Lower‐Extremity Functional Scale; NPRS, Numerical Pain Rating Scale; UEFI, Upper Extremity Functional Index.

* *p* < 0.050.

### Hierarchical multiple regression

The findings of each model are presented in Table 4. Participants' change in pain demonstrated a significant association with change on each functional outcome (Table 4). Models for the change in LEFS score, UEFI score, COPM‐Performance score, and COPM‐Satisfaction score overall explained 25.6%, 15.7%, 16.9%, and 13.4% of the variance in change in pain, respectively. Specifically, when adjusting for demographics, data source, and medical history, the participants' changes in LEFS score, UEFI score, COPM‐Performance score, and COPM‐Satisfaction score uniquely explained 19.8%, 7.8%, 12.0%, and 8.6%, of the variance in the change in pain, respectively.

**TABLE 4:** Regression models for change in pain.

	Model 1 (LEFS change)		Model 2 (UEFI change)	
	*B*	Sig.	*B*	Sig.
Intercept	1.46	0.209	3.77	0.011
Age	0.05	0.418	−0.03	0.697
Gender	−0.12	0.719	0.11	0.794
Hospital	−0.51	0.141	−0.71	0.070
History of injury	−0.75	0.011	−0.61	0.098
History of psych diagnosis	−0.68	0.021	−1.05	0.004
Functional metric	0.07	<0.001	0.05	<0.001
*R* ^2^	25.6*		15.7	
*R* ^2^ change				
Block I	0.3		0.1	
Block II	1.7*		2.8*	
Block III	4.5*		4.9*	
Block IV	19.1*		7.8*	

	Model 3 (COPM performance)		Model 4 (COPM satisfaction)	
	*B*	Sig.	*B*	Sig.
Intercept	1.66	0.178	1.86	0.150
Age	0.00	0.994	0.00	0.969
Gender	−0.17	0.628	−0.09	0.810
Hospital	−0.17	0.632	−0.19	0.615
History of injury	−0.61	0.049	−0.45	0.155
History of psych diagnosis	−0.90	0.004	−0.90	0.006
Functional metric	0.50	<0.001	0.32	<0.001
*R* ^2^	16.9*		13.4*	
*R* ^2^ change				
Block I	0.0		0.1	
Block II	0.9		0.9	
Block III	3.9*		3.9*	
Block IV	12.0*		8.6*	

*Note*: *R* ^2^ refers to the total variance explained by the model. *R* ^2^ change shows the variance explained by each block. Block I: age and gender (0 = Male; 1 = Female); Block II: Block I + Hospital (1 = CSH; 2  = RCH); Block III: Block II  +  history of injury (0 = no; 1 = yes) and history of psych diagnosis (0 = no; 1 = yes); Block IV (Model 1): Block III + LEFS scores; Block IV (Model 2): Block III + UEFI scores; Block IV (Model 3): Block III + COPM performance scores; Block IV (Model 4): Block III + COPM satisfaction scores.

Abbreviations: COPM, Canadian Occupational Performance Measure; LEFS, Lower‐Extremity Functional Scale; UEFI, Upper Extremity Functional Index.

* *p* < 0.050 for final block.

### ROC analysis

**FIGURE 1:**
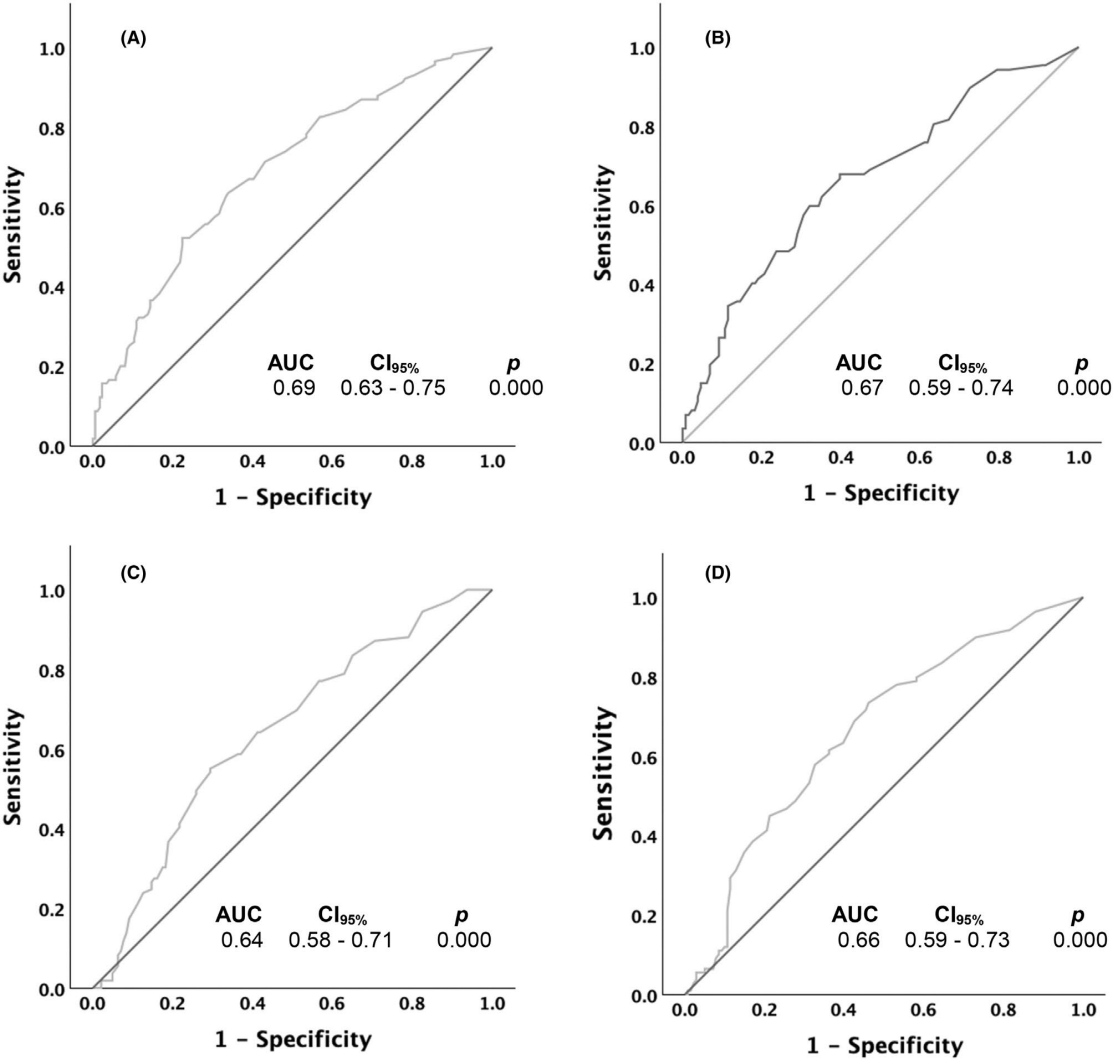
Receiver operating curves (ROC) used to calculate area under curve (AUC) and optimal cut‐points for (A) Lower Extremity Functional Scale, (B) Upper Extremity Functional Index, (C) Canadian Occupational Performance Measure—Performance, and (D) Canadian Occupational Performance Measure—Satisfaction.

Overall, 122 of the total 309 participants reported minimal change in pain (i.e., <30% change in pain). The ROC analysis revealed that participants' scores on the LEFS, UEFI, COPM‐Performance, and COPM‐Satisfaction scales at discharge were significantly able to distinguish between participant change in pain statuses (Figure 1).

Table 5 presents optimal thresholds for the LEFS, UEFI, COPM‐Performance, and COPM‐Satisfaction functional measures. The LEFS and COPM‐Performance measures had threshold values of 62.2 and 7.55, respectively, associated with higher specificity (i.e., ability of a test to rule‐in condition), 77.6% and 70.6%, respectively, compared to sensitivity (i.e., ability of a test to rule‐out a condition), 52.2% and 55.0%, respectively. Hence, both tests are good for ruling‐in risk of minimal change in pain following IIPT. In contrast, both UEFI and COPM‐Satisfaction had threshold values of 72.9 and 8.55, respectively, associated with higher sensitivity, 67.8% and 73.4%, respectively, compared to specificity, 60.3% and 53.9%, respectively. Hence, both tests are good for ruling‐out risk of minimal change in pain.

**TABLE 5:** Optimal cut‐points for functional measures to estimate change in pain status.

	LEFS (0–80)	UEFI (0–80)	COPM performance (0–10)	COPM satisfaction (0–10)
Cut‐point	62.2	72.9	7.55	8.55
Sensitivity	52.2	67.8	55.0	73.4
Specificity	77.6	60.3	70.6	53.9

Abbreviations: COPM, Canadian Occupational Performance Measure; LEFS, Lower‐Extremity Functional Scale; UEFI, Upper Extremity Functional Index.

## DISCUSSION

Children with moderate to severe chronic pain exhibit diminished physical function, likely due to persistent pain or behaviors stemming from fear‐related avoidance.^3^ IIPT programs focus on enhancing patient functioning with the overarching goal that this may lead to reducing perceived pain over time and with maintenance of pain management strategies.^9^, ^12^ Clinicians, however, face challenges in gauging post‐treatment pain changes, as they may not directly address pain relief during IIPT and instead focus on improving function despite pain. According to our findings, physical function and perceived pain are significantly associated, such that greater change in self‐reported physical function from admission to discharge is associated with greater change in perceived pain (Table 4). Furthermore, measures of self‐reported physical function such as LEFS, UEFI, COPM‐Performance, and COPM‐Satisfaction may significantly distinguish between a patient's change in pain status and thereby indicate their risk of experiencing minimal pain reduction post‐treatment (Figure 1; Table 5). These findings not only enhance the utility of these measures as outcomes of self‐reported physical function but also position them as potential clinical markers for screening changes in pain following IIPT, offering valuable insights for improved patient care.

Change in participants' lower and upper extremity function (per the LEFS and UEFI) was significantly associated with their change in pain (Table 4). This suggests that as children with chronic pain report improvements in their lower and upper extremity function, clinicians may anticipate a corresponding reduction in their perceived pain. Specifically, our analysis revealed every 1‐point change in LEFS or UEFI score was associated with a 0.07‐ or 0.05‐point reduction in pain. On average, participants in our study reported an increase of approximately 29 points on the LEFS and 20 points on the UEFI, consistent with prior research on IIPT (Table 2).^25^ Based on our findings, it may be inferred that this increase in LEFS or UEFI scores post‐treatment may correspond with an average reduction in pain of 2 or 1 point, respectively. These findings are further supported by previous research on adults with chronic pain, where strength training has been shown to improve muscle function and reduce perceived pain, particularly among individuals with fibromyalgia.^26^, ^27^, ^28^ Overall, findings underscore the importance of prioritizing functional enhancement as a strategy for alleviating perceived pain in individuals with chronic pain.

Personalized goal‐setting is an important aspect of patient rehabilitation, a pivotal process that empowers patients to identify functional tasks and activities vital to their recovery.^29^ According to our findings, change in pediatric patients' performance and satisfaction with their personalized goals (per the COPM) was significantly associated with their change in perceived pain. These findings underscore the importance of personalized goal attainment via functional improvement in alleviating pain following treatment. Specifically, for every 1‐point improvement in pediatric patients' scores on the COPM‐Performance or COPM‐Satisfaction scales, there was a corresponding reduction of 0.5 or 0.32 points in perceived pain. To contextualize these findings, participants in our study demonstrated an average increase of 3.9 and 5.0 points in their COPM‐Performance and ‐Satisfaction scores, respectively. Based on our analysis, it is likely that this improvement in COPM scores may correlate with a reduction of 2 or 1.5 points in perceived pain post‐treatment.

In addition to the observed relation between physical function and perceived pain, the identified thresholds for the physical function measures (Table 5), albeit preliminary, may allow clinicians to screen for the risk of minimal reduction in pain post‐treatment. Initial cut‐points for LEFS and COPM‐Performance demonstrated greater sensitivity than specificity, suggesting their potential utility in helping clinicians' rule‐in the risk of minimal reduction in pain following IIPT. For example, children with chronic pain scoring less than 62.2 on the LEFS and less than 7.55 on the COPM‐Performance measures are likely at risk of reporting minimal reduction in pain (i.e., less than 30%) following IIPT. Conversely, the preliminary cut‐points for UEFI and COPM‐Satisfaction exhibited greater sensitivity than specificity, suggesting their potential utility in helping clinicians' rule‐out risk of minimal reduction in pain following IIPT. For example, children with chronic pain scoring more than 72.9 on the UEFI and more than 8.55 on the COPM‐Satisfaction are likely to report moderate to substantial reduction in pain (30% or more) following treatment. Given IIPT may not directly address pain to prevent drawing the patients' attention toward pain, this approach provides an indirect means to assess change in pain levels. While these thresholds are derived from post‐discharge outcomes, clinicians may consider utilizing them at different time‐points during IIPT to gauge a child's progress toward achieving post‐treatment pain reduction. Further validation and refinement of these goals, however, is needed to enhance their clinical utility in estimating treatment outcomes and guiding individualized care strategies for children with chronic pain undergoing IIPT.

Physical function plays an integral role in a child's ability to engage in a wide range of tasks and activities, as well as participate in school and other social interactions.^30^ Patient rehabilitation within the framework of IIPT primarily focuses on restoring compromised functioning. While pain reduction is not the primary objective, clinicians anticipate a decrease in perceived pain alongside improved function.^9^, ^13^ This is crucial in preventing patients from experiencing relapses due to elevated pain levels post‐discharge from inpatient rehabilitation. Our study findings corroborate clinical observations regarding the intricate interplay between function and pain. However, the precise mechanisms underlying this relationship warrant further exploration. This understanding may empower clinicians to customize interventions and explore outpatient care or maintenance dosage options for children transitioning from inpatient care to their respective communities.

### Study limitations

Our study strengths include a relatively large and heterogeneous sample of pediatric chronic pain patients from two different regions of the country. This diversity enhances the generalizability; however, findings may only be limited to those who have attended inpatient IIPT programs. Additionally, given the cross‐sectional nature of study design, inferences on causal association between physical function and pain cannot be made. Future large‐scale longitudinal studies are necessary to determine the nature of relationship between enhanced functioning and reduced perceived pain. While preliminary cut‐points for estimating change in pain status through measures of physical function are provided, future studies may consider identifying cut‐points based on patient characteristics (e.g., pain diagnosis) or baseline function capabilities. Our findings are further limited by the absence of psychosocial measures, such as rate of anxiety or stress, which may need to be controlled in future investigations on this topic. Lastly, it's worth noting that our analysis is hindered by missing data, which may have resulted from various factors inherent to clinical datasets. This limitation necessitated a reduction in our sample size to those with complete data on most variables.

## CONCLUSIONS

Chronic pain poses a significant challenge to individuals' overall well‐being and functionality. IIPT for pediatric chronic pain patients aims to enhance physical function through a comprehensive approach. According to our findings, greater change in self‐reported physical function from admission to discharge is associated with a greater change in perceived pain. This suggests that measures of self‐reported physical function may serve as valuable indicators for clinicians to gauge post‐treatment pain reduction in children with chronic pain. Furthermore, our findings suggest that measures of self‐reported physical function may distinguish between children likely to experience minimal pain reduction (i.e., <30%) and those expected to achieve moderate to substantial pain relief (i.e., ≥30%). This nuanced understanding can empower clinicians to tailor treatment strategies more effectively to meet the individual needs of pediatric chronic pain patients. Future research may confirm findings in a larger sample of children with chronic pain while considering psychosocial and other potentially relevant factors. Further, longitudinal investigations may better highlight the relation between function and pain.

## AUTHOR CONTRIBUTIONS

All authors have contributed equally to the development of this manuscript.

## FUNDING INFORMATION

This research did not receive any specific grant from funding agencies in the public, commercial, or not‐for‐profit sectors.

## CONFLICT OF INTEREST STATEMENT

The authors have no conflicts of interest to report. The material within has been presented at the 2023 Annual Meeting of the American Congress of Rehabilitation Medicine, held from 30 October to 2 November 2023, in Atlanta, GA.

## REPRINTS

Reprints will not be available from the author.

## Data Availability

The research data for this manuscript is not available for sharing.
